# Evaluation of possible biomarkers for caries risk in children 6 to 12 years of age

**DOI:** 10.1080/20002297.2021.1956219

**Published:** 2021-08-17

**Authors:** María P Angarita-Díaz, Aurea Simon-Soro, Diana Forero, Felipe Balcázar, Luisa Sarmiento, Erika Romero, Alex Mira

**Affiliations:** a Department of Health Sciences, School of Dentistry, Universidad Cooperativa De Colombia, Villavicencio Campus, Colombia; b Department of Health and Genomics, Foundation for the Promotion of Health and Biomedical Research, Valencia, Spain; c Centre for Oral Health, School of Health and Welfare, Jönköping University, Sweden

**Keywords:** Biomarkers, child, dental caries, saliva

## Abstract

**Background:** Electrolytes, proteins, and other salivary molecules play an important role in tooth integrity and can serve as biomarkers associated with caries.

**Objective:** To determine the concentration of potential biomarkers in children without caries (CF) and children with caries (CA).

**Methods:** Unstimulated saliva was collected, and the biomarkers quantified in duplicate, using commercial Enzyme Linked Immunosorbent Assay (ELISA) kits to determine IgA, fibronectin, cathelicidin LL-37, and statherin levels, as well as colorimetric tests to detect formate and phosphate.

**Results:** Significantly higher concentrations of statherin was detected in the CF group (Median: 94,734.6; IQR: 92,934.6–95,113.7) compared to the CA2 group (90,875.0; IQR: 83,580.2–94,633.4) (p = 0.03). Slightly higher median IgA (48,250.0; IQR: 31,461.9–67,418.8) and LL-37 levels (56.1; IQR 43.6–116.2) and a lower concentration of formate were detected in the CF group (0.02; IQR 0.0034–0.15) compared to the group with caries (IgA: 37,776.42; IQR: 33,383.9–44,128.5; LL-37: 46.3; IQR: 40.1011–67.7; formate: 0.10; IQR: 0.01–0.18), but these differences were not statistically significant.

**Conclusion:** The fact that these compounds have been identified as good markers for caries among European adults highlights the difficulty of identifying universal biomarkers that are applicable to all ages or to different populations.

## Introduction

A biomarker is a characteristic that can be objectively measured and evaluated as an indicator of a normal biological process, a pathological process, or a response to a pharmacological treatment [1]. As indicators of a pathological process and depending on the case, these biomarkers allow the detection, characterization, monitoring or prognosis, and even provide information about the susceptibility or risk to develop a disease [2,3]. However, in order to identify valid biomarkers with some of these functions, it is important to understand the physiopathology and the factors associated to the disease, to subsequently identify the compounds that are activated or expressed before or during the progression of the illness. This will eventually help to determine if the biomarker shows a correlation with the pathological process [2].

Caries is the demineralization of dental tissue caused by organic acids produced during the metabolism of essentially cariogenic bacteria [4]. The prevalence and damage generated by these bacteria are influenced by host protective factors, such as immune competence, the production and buffering capacity of saliva, and hygiene and dietary habits [5]. Mechanisms have been sought to diagnose the risk of caries based on these factors using software such as Cariogram [6] or through the identification of biomarkers [7,8,9]. However, a significant amount of new studies are required to evaluate the validity of caries biomarkers, among other things because tooth decay is a multi-factorial disease with genetic, environmental and microbial effects playing a role, and because of a large inter-individual variability that renders the task of finding universal caries markers [10].

To identify biomarkers for dental caries, different biological samples have been tried, including saliva [11]. This fluid contains protective molecules that directly or indirectly influence oral microorganisms and their potential cariogenic effect, thus allowing for the risk of caries to be predicted [11]. These molecules include those related to the immune system such as immunoglobulins, antimicrobial peptides, and proteins of the complement system [12]. For example, IgA and cathelicidin LL-37 concentrations have been shown to be related to oral health [13,14]. IgA acts as the first immunological defence agent on the surface of the oral mucosa and teeth because it interferes with bacterial colonization and neutralizes toxins, viruses, and other types of microorganisms [14]. Cathelicidin LL-37 is a peptide involved in several functions such as antimicrobial functions, healing, and angiogenesis. Regarding the microbial control function, this peptide belongs to the amphipathic cationic family, which is produced by multiple cells. Moreover, they belong to the first line of defence against pathogens such as Gram-negative and Gram-positive bacteria and *Candida albicans* [13].

Other molecules that can possibly be used as biomarkers are those related to the adhesion capacity of microorganisms, including components that control the attachment of microorganisms to the surface of the teeth such as fibronectin and statherin [15]. Fibronectin is a glycoprotein with multiple functions in different biological processes such as adhesion to the extracellular matrix, differentiation, cell growth and migration, embryonic development, and wound healing. In addition, it plays a role in antimicrobial defence [15], and interferes with the colonization of microorganisms by blocking the adhesion of bacteria present in the oral cavity such as *Streptococcus mutans* and *Streptococcus mitis* [16]. Statherin is a 43-amino acid and low-molecular-weight peptide (5.4 kDa) with multiple functions, such as protecting the surface of the teeth by trapping calcium ions, which promotes remineralization [15]. Furthermore, it plays a role in microbial control by reducing bacterial and fungal colonization through the aggregation of microorganisms, thus hindering their capacity to adhere to hard tissue and epithelium [15].

Finally, there are pH-related molecules, such as those that contribute to acidification [17] or those that, conversely, neutralize this acidification of saliva and dental biofilm [18]. The first group includes organic acids produced by bacteria such as lactic acid, propionic acid, acetic acid, and formic acid [19]. These acids circulate through the dental plaque towards the porous enamel, dissociating and releasing hydrogen ions, which quickly dissolve the enamel mineral. This leads to the demineralization or loss of calcium and phosphate [17,19]. Phosphate is among the second group of molecules that contribute to the neutralization of acids and, along with calcium, modulates the demineralization and remineralization process of teeth to prevent caries and dental erosion. Several studies demonstrate that these ions are decreased in the saliva of patients with active carious lesions. Therefore, this biochemical parameter could play an important role in determining individual caries and the susceptibility to demineralization of other teeth [18].

[7], analysed 30 biomarkers belonging to these three categories (immune, adhesion, and pH) in the saliva of adult Spanish patients between 19 and 39 years old and identified molecules that maximize the diagnostic separation of health and caries conditions, which included two immune related compounds (cathelicidin LL-37 and IgA), two related to adhesion capacity (statherin and fibronectin), and two pH-related (phosphate and formate). The present study included a pilot test to evaluate the differential levels of these biomarkers in the saliva of 6- to 12-year-old children from Villavicencio, Colombia, with and without caries, and to analyse whether these biomarkers can be useful in children from populations other than the Spanish one.

## Materials and methods

### Sample and selection criteria

A pilot study approved by the Ethics Subcommittee of Universidad Cooperativa de Colombia (No. 009–2017) was conducted where unstimulated saliva was collected from children 6 to 12 years old, without systemic diseases, without appliances in the mouth, and without antibiotic treatment over the last three months, who had previously provided informed consent and assent. The parents or caregivers of the participating children completed a questionnaire on oral hygiene and dietary habits before sample collection. An International Caries Detection and Assessment System (ICDAS)-certified dentist (kappa value ≥ 0.7) determined the dental plaque index using the modified Silness–Löe index, through an intraoral examination, and established the child’s caries diagnosis as per ICDAS criteria. Previous work studying these biomarkers in adults showed that ten individuals per group were sufficient to detect statistically significant differences [7]. We therefore aimed at recruiting ten CF individuals and an equivalent sample size from each of the caries groups. After recruitment, samples of 12 caries-free children (CF), 9 children with ICDAS-1 and 2 caries (CA1), and 12 children with ICDAS > 3 (CA2) were analysed in this study. The ICDAS diagnosis was determined after collecting the saliva sample and dental prophylaxis.

### Sample collection and analysis

A sample of 2 mL of unstimulated saliva was collected between 8 and 11 am, after the children had brushed their teeth with water only, and 10 min after a 1-minute mouthrinse with a 10% sugar solution. The sugar rinse was performed to maximize the presence of organic acids within the sample, and therefore increase their possible discriminatory power. This rinsing did not affect the levels of the other biomarkers in the study performed by [7]. Saliva samples were frozen and transported to the Genomics and Health Laboratory of FISABIO, Spain, where they were analysed according to a previously standardized technique.

### Quantification of the molecules to be evaluated

The analyses were conducted in duplicate for biomarker quantification, using commercial kits of Enzyme-Linked Immunosorbent Assays (ELISA); Assaypro (Saint Charles, MO) was used for secretory IgA (Sample dilution: 1:1,000) and fibronectin (1:20), HycultBiotech (Uden, North Brabant, Netherlands) for cathelicidin LL-37 (1:200), and Cloud Cone Corp (Houston, TX) for statherin (1:200). Finally, BioVision colorimetric tests (Milpitas, CA) were applied to quantify formate (1:5) and phosphate (1:200). Analyses were then performed according to the manufacturer’s recommendations.

### Data analysis

The SPSS 25.0 software package was used for data analysis. Descriptive analyses were conducted, and the normality of the data and homogeneity of variances were determined. The Mann–Whitney U test was performed to compare the groups without caries and with caries to determine the statistical differences in biomarker expression and groups of children. The Kruskal–Wallis test was performed to compare the CF, CA1, and CA2 groups. A post-hoc test was performed with Kruskal–Wallis in the case of statherin. The collected data set was cleaned prior to analysis to detect and treat lost data and aberrant or atypical values.

## Results

### Characteristics of the population

The average age of the population was 8.33 ± 1.71 years, and 58% of the children were female (Table 1). Moreover, 36.4% of the children were healthy or caries-free (CF) and 63.6% had caries (Caries Group, or CA). CA children were distributed as follows: 18.1% had ICDAS 1, 9.1% had ICDAS 2 (CA1), 3% had ICDAS 3, 9.1% had ICDAS 5, and 24.2% had ICDAS 6. A group with ICDAS > 3 was formed (CA2). Most children participating in the study presented poor oral hygiene (51.5%), followed by moderate hygiene (39.4%); this distribution was similar between groups. Moreover, the children in the CF group had better oral hygiene and a lower average dental plaque index (31.1 ± 15.3) compared to the other groups without showing significant differences (p = 0.83) (see Table 1 for details).

**Table 1.** Characteristics of the children participating in the study

Plaque index	Caries Free	Caries Group 1	Caries Group 2	p-value
Mean, Standard deviation	31.1 ± 15.3	33.3 ± 7.7	35.9 ± 22.7	0.83^a^
Oral Hygiene				
Good	16.7%	0	8.3%	0.63^b^
Moderate	33.3%	44.4%	33.3%	
Poor	50%	55.6%	58.3%	
Brushing teeth with fluoride toothpaste				
>2 times per day	91.7%	100%	83.3%	0.42 ^b^
< 2 times per day	8.3%	0%	16.7%	
Number of meals and/or drinks per day				
>7 times per day	0	88.8%	0%	0.25 ^b^
< 7 times per day	100%	11.1%	100%	

^a^After confirming normality of the data and homogeneity of the variances, the value was calculated with the ANOVA test. ^b^Value calculated with the chi-square test. No significant differences were detected (p > 0.05).

Furthermore, most children brushed their teeth with fluoride toothpaste more than two times a day, and the percentage of those who did not in CA2 was higher, although not significant. The frequency of meals and/or drinks, including fermentable carbohydrates was lower than seven times a day in the CF and CA2 groups (Table 1).

### Molecules associated with the immune system

**Figure 1.**
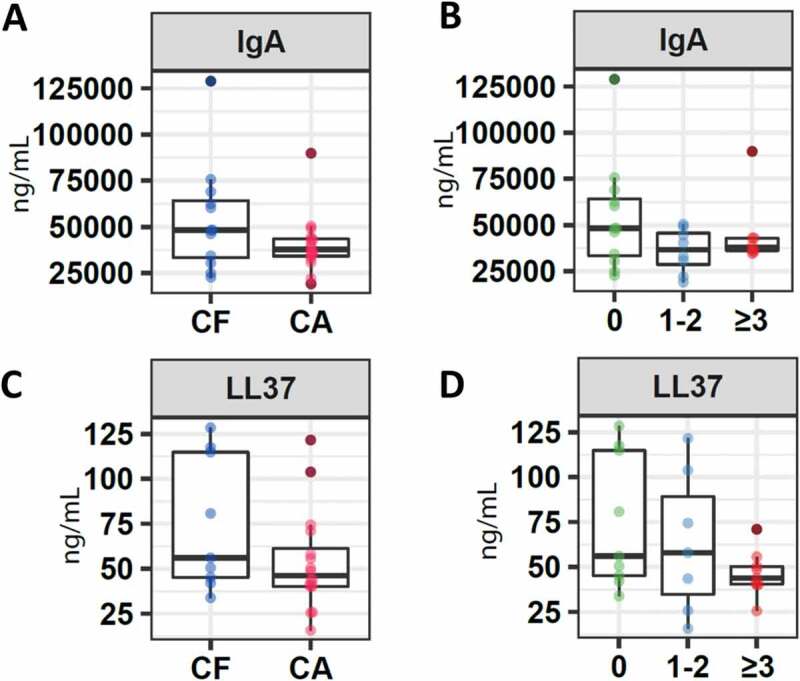
Salivary concentration of molecules associated with the immune system. Box plots show: **A**. Levels of IgA in children without caries (CF) and with caries. **B**. Levels of IgA in children without caries (CF), children with ICDAS 1 and 2 (CA1), and children with ICDAS greater than 3 (CA2). **C**. Levels of cathelicidin LL-37 in children without caries (CF) and with caries. **D**. Levels of cathelicidin LL-37 in children without caries (CF), children with ICDAS 1 and 2 (CA1), and children with ICDAS greater than 3 (CA2)

The concentrations of molecules associated with the immune system, such as IgA and cathelicidin LL-37, were higher in the group of children without caries with a median for IgA of 48,250.0 (IQR 31,461.9–67,418.8) and for LL-37 of 56.1 (IQR 43.6–116.2), as opposed to the group of children who presented a certain index of caries, with a median for IgA of 37,776.42 (IQR 33,383.9–44,128.5) and for LL-37 of 46.3 (IQR 40.1011–67.7) (Figure 1A and 1C). However, these differences were not statistically significant (IgA p-value = 0.12, LL-37 p-value = 0.56) (Figure 1A and 1C).

Comparing the concentration of these molecules based on the distribution of the three groups determined that a higher concentration of IgA was reported in the CF group, compared to CA1 (36,652.6 IQR 24,242.4–47,971.6) and CA2 (37,776.4 IQR 35,583.6–43,012.3), with no significant differences (p = 0.32). A slightly higher value was detected in CA1 (57.9 IQR 25.8–103.8) compared to CA2 (43,9 IQR 40.2–53.0) (p = 0.21) in the case of the LL-37 molecule (Figure 1B and 1D).

### Adhesion molecules

**Figure 2.**
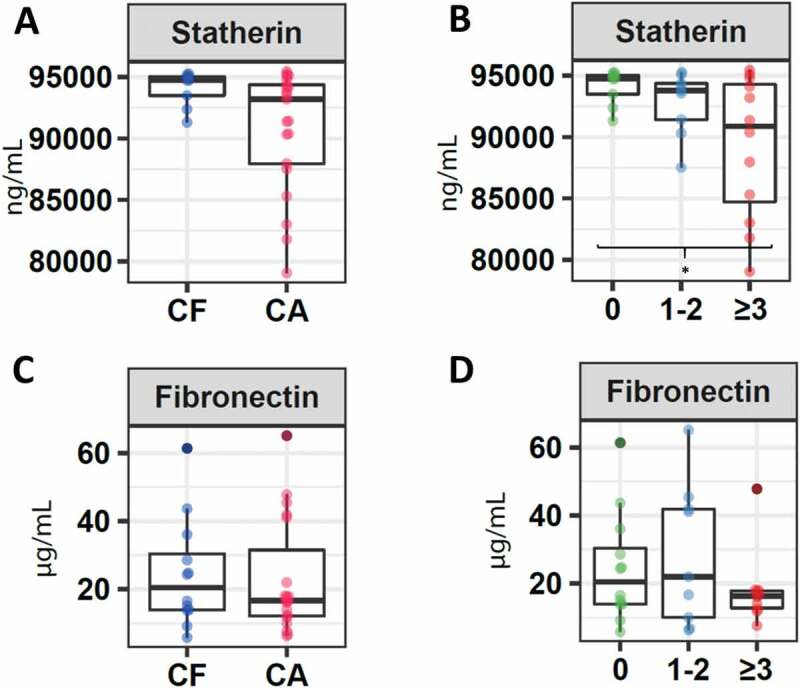
Salivary concentration of molecules associated with the adhesion capacity of microorganisms. Boxplots correspond to: **A**. Levels of statherin in children without caries (CF) and with caries. **B**. Levels of statherin in children without caries (CF), children with ICDAS 1 and 2 (CA1), and children with ICDAS greater than 3 (CA2). **C**. Levels of fibronectin in children without caries (CF) and with caries. **D**. Levels of fibronectin in children without caries (CF), children with ICDAS 1 and 2 (CA1), and children with ICDAS greater than 3 (CA2). The asterisk (*) indicates significance of p < 0.05 as per the Kruskal–Wallis analysis

Regarding adhesion molecules, a higher concentration of statherin was detected in children belonging to the CF group (94,734.6 IQR 92,934.6–95,113.7), compared to children who presented a certain index of caries (93,199.1 IQR 87,737.9–94,587.9) (p = 0.3). Furthermore, a significantly lower concentration of this molecule was detected in CA2 children (90,875.0 IQR 83,580.2–94,633.4), compared to CF children (p = 0.03) (Figure 2A and 2B). Regarding fibronectin, a slightly higher concentration was reported in CF patients in this investigation (CF: 20.43 IQR 13.8–34.2) compared to children with certain level of caries (16.7; IQR 11.9–41.1) (p = 0.7), being lower in children with a higher caries index (CA2 16.2 IQR 12.3–17.9) (Figure 2C and 2D), although no statistically significant differences were detected between these three groups (p = 0.7).

### Molecules associated with pH

**Figure 3.**
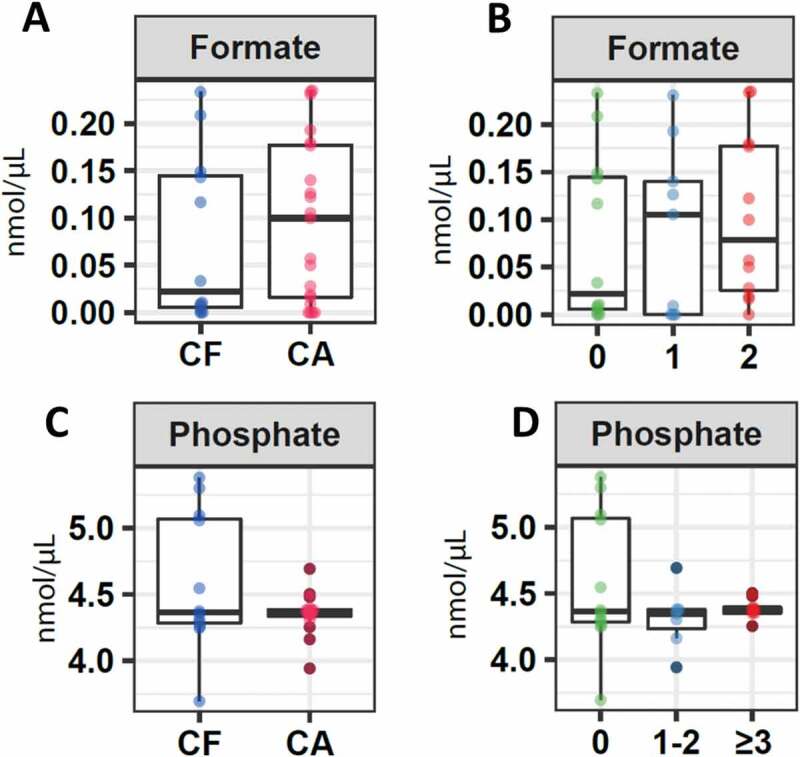
Salivary concentration of molecules associated with the pH. Boxplots represent: **A**. Differential levels of formate in children without cavities (CF) and with cavities. **B**. Formate levels in children without caries (CF), children with ICDAS 1 and 2 (CA1), and children with ICDAS greater than 3 (CA2). **C**. Phosphate levels in children without cavities (CF) and with cavities (C). **D**. Phosphate levels in children without caries (CF), children with ICDAS 1 and 2 (CA1), and children with ICDAS greater than 3 (CA2)

CF patients presented a lower concentration in the median of formate (0.02; IQR 0.0034–0.15) compared to the group of children who presented a certain index of caries (0.10; IQR 0.01–0.18), although no significant differences were reported (p = 0.9) (Figure 3A). A lower but not significant concentration was also detected (p = 0.6) when comparing the CF with the GA1 (0.11; IQR 0.00–0.17) and GA2 (0.08; IQR 0.005–0.18) groups (Figure 3B).

Regarding phosphate, the median value was very similar between the CF and the caries groups (CF: 4.37; IQR 4.27–5.09; CA: 4.37; IQR 4.32–4.43) (Figure 3C). The same was detected when the CF group was compared to the other two groups: CA1 (4.36; IQR 4.19–4.61) and CA2 (4.38; IQR 4.35–4.44) without detecting significant differences (p = 0.86) (Figure 3D).

## Discussion

Saliva is an essential protective factor in preventing tooth decay because of components such as ions that prevent demineralization and molecules with antimicrobial effect or that interfere with the colonization of microorganisms [8]. There are multiple studies that compare the concentration of these components in patients with caries and without caries to define the types of molecules that indicate a possible risk of caries [7, 20–24]. In this investigation, six types of molecules, which were selected according to the results of the investigation by [7], were analysed.

Among the six molecules analysed, the expression of the statherin molecule was significantly higher (p < 0.05) in the group of caries-free children compared to the children who presented the highest caries rates (ICDAS > 3). Moreover, the concentration of this molecule decreased as the severity of the caries index increased. Different authors have demonstrated an important association between the absence of caries and presence of this molecule. They include [25], who reported a strong correlation between this peptide and the absence of the disease from a liquid chromatography analysis [HPLC-MS] performed on the saliva of 20 children free of caries and with susceptibility to caries. [7], reported a significantly higher concentration of this molecule in 20 adult patients (19–39 years old), under healthy conditions compared to patients who presented caries (p < 0.05). In another study, [26], analysed the concentration of statherin in enamel pellicle collected from patients with dental erosion compared to healthy patients with an age range of 18–65 years (n = 60). The authors reported a significantly lower concentration in areas affected by dental erosion (p < 0.05). This result was similar to that of a more recent study where [27], compared the statherin concentration of the enamel pellicle collected from healthy surfaces with eroded surfaces in 29 patients (age range = 25–61 years). Therefore, an increasing number of studies have demonstrated the protective role of this molecule because of the regulation in the trapping of Ca and P ions to the hydroxyapatite of the tooth, and due to the reduction in the adhesion capacity of bacteria and fungi to oral surfaces [28,29]. This occurs through the agglutination of these microorganisms or through the inhibition of the adhesion of other molecules to the teeth, as it was demonstrated in an *in vitro* study by [30]. These authors used the stimulated saliva of a 30-year-old patient, which was fractionated by absorption to hydroxyapatite and then filtered with chromatography to isolate the proteins present and thus conduct bacterial adhesion tests. The authors demonstrated that salivary statherin and histatin inhibit the absorption of a fraction of a high-molecular-weight salivary glycoprotein isolated in the study, thus reducing the absorption of *S. mutans* bacteria on the hydroxyapatite surface.

Regarding the results of IgA, this study detected a non-significant higher concentration in healthy children. Studies performed to compare the concentration of these molecules in saliva have demonstrated an association between higher concentration levels and the absence of caries, for example, in the studies performed by [20], Omar et al. [21] and Razi et al. [31], who analysed the presence of IgA in the saliva of children between 3 to 8 years of age [20,21] and adolescents between 12 and 15 years [31]. In addition, a higher proportion of IgA-coated bacteria was found in the saliva of caries-free compared to caries-active individuals [32], suggesting a protective role of this antibody. However, other studies reported a higher concentration of IgA in children and adolescents between 3 and 16 years of age with caries [23], suggesting that other factors, in addition to caries, may affect the levels of this immunoglobulin.

Although the LL-37 concentration was higher in the healthy patients analysed in this study, the difference was not significant [33], reported a significantly higher concentration in the saliva of 49 healthy children and young people, between 2 and 18 years of age, compared to those with high cariogenic activity, thus concluding its possible protective role against caries. However, there are other authors who do not report significant differences in the expression of this molecule in both conditions such as [12], who analysed samples of unstimulated saliva collected from 149 healthy children and young people between 11 and 15 years of age with different indices of caries; [34] in saliva samples of 106 children between 10 and 71 months, and [7], in samples of saliva from adult patients under the same collection conditions as the present study, i.e. unstimulated saliva samples collected after 10 min of rinsing with 10% sugar solution. There are reports of a higher concentration of this molecule under caries conditions such as that of [22], who determined a slight positive relationship between LL-37 and the presence of caries in children between 36 and 60 months, and [7], with samples from adult patients without any rinsing with sugar solution before taking the samples.

Our investigation reported a slightly higher concentration of fibronectin in the caries-free patients. Furthermore, it was detected that the concentration was lower in the group with the highest caries index (ICDAS >3), although no significant differences were reported. This study was similar to that of [7], performed in healthy adults compared to those who had caries after rinsing with a sugar solution. There are studies that suggest the role of soluble fibronectin present in saliva in agglutinating microorganisms, thus reducing microbial adhesion to dental tissue [35]. Another study demonstrated an inverse relationship between the presence of soluble fibronectin and the quantification of *S. mutans* in the saliva of 12-year-old children with different caries indices [16].

Regarding the molecules associated with pH, this study reported that the median of formate was lower in caries-free children. The work by [7], detected a significantly higher expression of formate in adults who presented caries compared to healthy individuals after rinsing with a sugar solution. There are several studies that show a relationship between an acidic pH and presence of caries such as the one performed by [36], with 90 children between 26 and 70 months and [37], with 75 school children between 4 and 12 years old. This is because of the production of organic acids released by the microorganisms present in dental biofilm or saliva [19,38]. However, the interactions between different organic acids are difficult to evaluate and can be synergistic in their demineralization potential [39], and therefore the use of a single acid as a biomarker may be misleading.

Regarding phosphate, we did not observe significant differences in caries-free patients compared to those with a certain index of this disease. Other investigators have reported a slightly higher expression in caries-free children compared to those with this disease. These authors include [40], who analysed saliva samples from 75 children between 3 and 5 years of age, [31], in saliva from 40 children and adolescents, and [41], in 90 children from 3 to 6 years of age. [7], detected a significantly higher expression of this component in caries-free adults after rinsing with a sugar solution. However [36], reported higher levels in saliva samples from children with caries compared to healthy children.

Sample size was among the limitations of this study, and could have affected the statistical power of the comparisons between caries-free and caries-experienced groups. Therefore, although the difference between CF and CA individuals in our data was noteworthy, most of the compounds showed a high degree of inter-individual variability and produced no significant differences. Thus, analyses with a more representative sample size are recommended to contribute to the establishment of robust biomarkers for caries risk across populations. Furthermore, this study shows that biomarkers that may be valid in one population may not be valid in another with different geographic, genetic, or dietary characteristics. Similarly, certain molecules that acquire stable levels in an adult population can still be under development in children, such as immunological parameters, and therefore lose discriminatory value. Finally, caries is clearly a multifactorial disease, influenced by external factors such as diet or oral hygiene, microbiological factors such as the levels of cariogenic bacteria, and host-related factors, included those studied in this investigation (levels of proteins and salivary metabolites). It is probable that individuals at risk for caries because of inappropriate levels of certain protective or caries-promoting molecules do not develop the disease because of other external factors such as brushing or low levels of sugar in the diet that counteract this intrinsic tendency [42]. In this sense, certain children sampled in this study who did not have caries at the time of sampling (CF) could develop caries in the future, thus highlighting the importance of longitudinal studies.

## Conclusion

Among the results of this study, a significantly higher expression of statherin was detected in saliva samples collected from healthy children compared to those collected from the group with moderate and severe caries [ICDAS > 3). Regarding other molecules, no significant differences were detected between groups. This lack of significance may be attributed to low sample size and to the absence of the discriminatory value of these molecules among the child population, in contrast to adults. In addition, it may also reflect the requirement to include additional parameters such as dietary features and/or oral hygiene habits to obtain a more robust global estimate of the risk for caries.
